# Turmeric Induced Liver Injury: A Report of Two Cases

**DOI:** 10.1155/2019/6741213

**Published:** 2019-04-28

**Authors:** Raphael P. Luber, Clarissa Rentsch, Steve Lontos, Jeffrey D. Pope, Ar Kar Aung, Hans G. Schneider, William Kemp, Stuart K. Roberts, Ammar Majeed

**Affiliations:** ^1^Department of Gastroenterology, The Alfred Hospital, 55 Commercial Road, Melbourne, Victoria, Australia; ^2^Department of Gastroenterology and Liver Transplantation, The Austin Hospital, 145 Studley Road, Heidelberg, Melbourne, Victoria, Australia; ^3^Central Clinical School, Monash University, The Alfred Hospital, 99 Commercial Road, Melbourne, Victoria, Australia; ^4^Department of Pharmacy, The Alfred Hospital, 55 Commercial Road, Melbourne, Victoria, Australia; ^5^Clinical Biochemistry Unit, The Alfred Hospital, 55 Commercial Road, Melbourne, Victoria, Australia; ^6^Department of Forensic Medicine, Monash University, Melbourne, Australia; ^7^Department of Epidemiology and Preventive Medicine, Monash University, Melbourne, Australia; ^8^Department of General Medicine, The Alfred Hospital, 55 Commercial Road, Melbourne, Victoria, Australia

## Abstract

Turmeric is a commonly used oral herbal supplement with purported anti-inflammatory and antineoplastic properties. It is promoted as safe, with limited reports of severe adverse effects directly related to oral turmeric thus far in the literature. Herein we report two cases of turmeric supplement induced severe hepatitis. These cases highlight the need for physicians to be aware of patients taking this common supplement and the potential risks that exist.

## 1. Introduction

Idiosyncratic drug induced liver injury (DILI) is estimated to occur in 1 in 10,000 to 1 in 100,000 people who take a medication [1]. It is the most frequent adverse event leading to abandonment of new drugs and is implicated in 13% of acute liver failure cases in the United States [2]. Herbal and dietary supplements (HDS) account for at least 9% of cases overall [1], with this rate thought to be an underestimate due to their popularity and underreporting.

Turmeric, with its major active ingredient curcumin, is one of the most commonly used HDS [3]. In recent years it has gained attention for its anti-inflammatory, anticancer, and other properties across a variety of disorders [4]. Regarding safety, dosing studies and randomized controlled trials have thus far concluded that curcumin treatment is safe, with minimal reported severe adverse effects [4–7].

We report two cases of likely turmeric supplement induced severe liver injury, including one in which a positive rechallenge occurred.

## 2. Case 1

A 52-year-old Caucasian female presented to her general practitioner with a one-week history of nausea, pruritus, and painless jaundice with associated pale stools and dark urine. This occurred approximately one month following commencement of a turmeric supplement among other medications. She rarely consumed alcohol, was a nonsmoker, and had no history of tattoos, illicit drug use, or recent travel. She had no prior history of liver disease and had normal liver function tests three months before. Her medical history was notable only for oligoarticular osteoarthritis.

**Figure 1:**
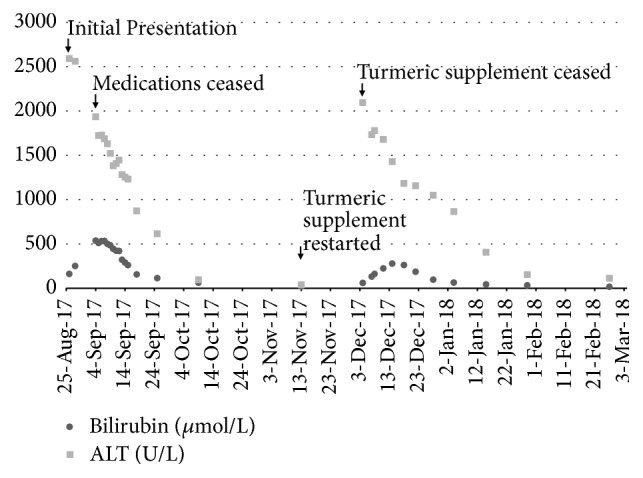
Bilirubin and ALT trend in relation to turmeric supplement use in Case 1.

On presentation she was found to have a bilirubin of 162 *μ*mol/L with a hepatocellular profile on liver function tests (ALT 2591U/L, AST 1770U/L, ALP 263U/L, and GGT 370U/L) and preserved hepatic synthetic function (INR 1.0, albumin 32 g/L) (Figure 1). She was jaundiced, with no hepatomegaly or clinical features of chronic liver disease on examination. With progressive jaundice over the subsequent days she was referred to the emergency department, at which point her bilirubin peaked at 536 *μ*mol/L.

Approximately one month prior to presentation she had commenced one tablet per day of Ancient Wisdom Modern Medicine® High Potency Turmeric (375mg curcuminoids and 4 mg black pepper per tablet), along with a flaxseed oil supplement and occasional diclofenac use for arthritic pain. Her long-term medications of cholecalciferol 50mcg daily and ascorbic acid 500 mg daily along with a levonorgestrel 52 mg intrauterine device had been unchanged for at least a year. There was no recent history of paracetamol use.

Upon admission, all oral medications and supplements were ceased. Apart from a detectable hepatitis B core antibody (surface antigen negative, surface antibody positive, and HBV viral load, not detectable), the remainder of this screen was unremarkable with negative hepatitis A, C, and E serology, negative CMV, EBV, VZV, and HSV IgM and PCR, negative liver-kidney, mitochondrial, smooth muscle, antinuclear, anti-tissue transglutaminase and anti-deamidated gliadin antibodies. Plasma eosinophil count, serum copper, and ceruloplasmin were all within normal limits, and paracetamol level was undetectable. Abdominal ultrasonography showed patent portal and hepatic veins with no biliary duct dilatation.

Due to lack of significant improvement by day four of admission (bilirubin 534*μ*mol/L, ALT 1686U/L, ALP 252U/L, and GGT 400U/L) a liver biopsy was performed. Histology showed nonspecific inflammatory changes with generally preserved hepatic architecture and no fibrosis. A florid inflammatory cell infiltrate comprising lymphocytes, histiocytes, neutrophils, scattered eosinophils, and minimal plasma cells were noted in the portal tracts with associated interface hepatitis and reactive changes in bile duct epithelium. A drug reaction was the favored differential.

She was discharged day 12 of admission (bilirubin 260*μ*mol/L, ALT 1232U/L) with the presumptive diagnosis of diclofenac induced liver injury. By two months after admission her LFTs had normalized (bilirubin 21*μ*mol/L, ALT 33U/L) and she was discharged from the clinic.

At this point she recommenced the turmeric supplement (1125mg curcuminoids per day) as sole therapy for her arthritis. Three weeks later her nausea recurred and repeat liver function tests showed an acute hepatitis (ALT 2093U/L, AST 1030U/L, and bilirubin 60*μ*mol/L). Repeat viral serology was unremarkable. She was advised to cease the turmeric supplement, and two months later her liver function tests had again normalized. The calculated Roussel UCLAF causality assessment method (RUCAM) score pertaining to the causal implication of the turmeric supplement was 9 or “highly probable”, augmented by the rechallenge [8].

The turmeric supplements were sent for analysis. A sample of the supplement was pulverized, dissolved in methanol to a concentration of 1 mg/mL, and filtered through a 0.45 *μ*m polyvinylidene fluoride filter. The filtrate was diluted and analyzed by a validated liquid chromatography quadruple time-of-flight (LC-QTOF) mass spectrometry method. Results were compared to a toxicology library containing approximately 1400 compounds, including medications, illicit drugs, and over-the-counter medicines. A detailed method is published elsewhere [9]. A further sample was analyzed by inductively coupled plasma (ICP) mass spectrometry for the presence of trace and toxic elements. The turmeric supplement tested negative for drugs, adulterants, or toxic heavy metals.

## 3. Case 2

A 55-year-old man of Italian background presented to his general practitioner for a routine checkup and was found to have an asymptomatic transaminitis on blood panel (ALT of 1149U/L, bilirubin 23*μ*mol/L, ALP 145U/L, and GGT 302U/L). He occasionally drank alcohol, was a nonsmoker, and had no recent travel history or risk factors for viral hepatitis. Examination was unremarkable. His background history included idiopathic thrombocytopenic purpura, hypertension, gout, and osteoarthritis, with regular medications including long-term Telmisartan, Atenolol, and Lercanidipine. He had no known liver history with normal liver function tests one year prior. His only new medication was commencement of a turmeric supplement five months prior.

He was referred to a hepatologist and underwent a screen for causes of acute hepatitis. Apart from a positive ANA titre of 1:160, screening was unremarkable. Hepatic synthetic function was preserved with a normal INR and albumin of 45 g/L. Abdominal ultrasonography showed diffuse steatosis, but no ductal or vascular pathology. A drug reaction was suspected, and the turmeric supplement was ceased.

Close follow-up occurred over the subsequent four months. Near normalization of liver function tests occurred by one month (ALT 96U/L, bilirubin 10*μ*mol/L) with further improvement by four months after cessation (ALT 46U/L, bilirubin 11 *μ*mol/L). The turmeric supplement was the presumed cause of the hepatitis and as such the patient was not rechallenged. The RUCAM score was 6, or “probable” [8]. The turmeric supplement was not known, and therefore further analysis could not be performed on the supplement.

## 4. Discussion

Turmeric rhizome has been used for thousands of years as a spice, a dye and for its purported medicinal properties. An increasing body of* in vivo* data and emerging clinical evidence exists for the anti-inflammatory, antioxidant, immunomodulatory, wound healing, antiproliferative, and antimicrobial activities of its major active curcuminoid constituent, curcumin, across a number of conditions [4, 10].

Products containing turmeric are generally listed by the curcuminoid content, commonly making up only 3-5% of turmeric rhizome and giving turmeric its yellow appearance [10]. Curcumin exhibits poor bioavailability, undergoing extensive first pass metabolism with doses of at least 4 g per day required for detectability in plasma [5, 6]. Formulation modifications to improve bioavailability include the use of liposomal encapsulation, nanoparticles, emulsions, and sustained released preparations [10–12]. When delivered with the alkaloid piperine, derived from* piper nigrum* (black pepper) and other piper species, plasma levels of curcumin have been shown to be augmented in humans and rats due to increased intestinal absorption and inhibition of hepatic glucuronidation by piperine [13].

Paradoxically to the presented cases, curcumin has been studied for its benefits in a number of hepatic pathologies. In humans, a combination formula of 1 g each of curcumin and* Tinospora cordifolia* has been shown to reduce hepatotoxicity in patients with active tuberculosis undergoing antituberculous treatment [14]. In another human pilot study, patients with nonalcoholic fatty liver disease randomized to 70 mg curcumin daily for 8 weeks displayed a significant reduction in sonographic liver fat content compared to placebo [15].

Regarding adverse effects, a number of severe adverse effects have been reported when given orally [16–18], topically [19], vaginally [20], and intravenously [21]. Mild liver function test derangement has been reported in approximately 5% of cases in randomized controlled trials [22], with one report of drug induced autoimmune hepatitis reported recently [22]. The potential for drug interactions has been recognized, with dose-dependent inhibition of cytochrome p450 subtypes CYP3A4 and CYP1A2 in the liver [23] and intestines [24] identified. Accordingly, the addition of curcumin to medications metabolized by CYP3A4 can lead to an increase in plasma levels, with curcumin associated with acute calcineurin inhibitor nephrotoxicity due to CYP3A4 inhibition [25].

However, while clinical studies may assess pure curcumin compounds, the products available to patients in pharmacies and nutritional food stores contain varying concentrations of curcumin plus numerous additives, potentially accounting for differing real-life risk profiles [26]. In case 1, the supplement used contained 4 mg of piperine in addition to 375 mg of curcumin. A dose of 20 mg of piperine in a similar ratio to curcumin has been shown to increase curcumin bioavailability 20-fold compared to curcumin alone due to inhibition of first pass metabolism in the intestines and liver [13]. Given the known pharmacological mechanisms of piperine and lack of any reported direct adverse effects in the literature, the hepatotoxicity in case 1 is felt more likely to be due to augmented effects of curcumin than the piperine itself. Furthermore, no other known toxic substances or toxic levels of heavy metals were found in the supplement on LC-QTOF and ICP mass spectrometry, respectively, essentially excluding a contaminant albeit for rare, unknown compounds. The curcumin supplement used in case 2 is not known, and hence comment on additives cannot be made.

The presented cases are “highly probable” and “probable,” respectively, for turmeric induced hepatotoxicity based on RUCAM scores. In both cases the timing of turmeric supplement use with respect to onset and offset of liver injury was compatible, and both underwent extensive serological screening excluding other causes of deranged liver function. The rechallenge in case 1, along with the histology consistent with drug toxicity and lack of any other known toxins on further analysis of the supplement, provides an even stronger case for the turmeric inducing hepatotoxicity and negates any relationship between other medications and the liver injury.

With high community interest in HDS therapies and emerging evidenced based support for turmeric across specialties, there will likely be increasing use by patients. In this context, the reported cases of turmeric induced hepatotoxicity highlight the need for the physician to be aware of patients taking this therapy and the potential risks that exist.
